# A critical analysis of the decreasing trends in tuberculosis cure indicators in Brazil, 2001-2022

**DOI:** 10.36416/1806-3756/e20240018

**Published:** 2024-05-08

**Authors:** Gabriel Pavinati, Lucas Vinícius de Lima, Pedro Henrique Paiva Bernardo, Jhenicy Rubira Dias, Bárbara Reis-Santos, Gabriela Tavares Magnabosco

**Affiliations:** 1. Programa de Pós-Graduação em Enfermagem, Universidade Estadual de Maringá, Maringá (PR) Brasil.; 2. Programa de Residência em Enfermagem, Universidade Estadual de Londrina, Londrina (PR) Brasil.; 3. Rede Brasileira de Pesquisa em Tuberculose - Rede TB - Rio de Janeiro (RJ) Brasil.

**Keywords:** Tuberculosis, Time factors, Retrospective studies, Brazil

## Abstract

**Objective:**

To analyze the temporal trend of tuberculosis cure indicators in Brazil.

**Methods:**

An ecological time-series study using administrative data of reported cases of the disease nationwide between 2001 and 2022. We estimated cure indicators for each federative unit (FU) considering individuals with pulmonary tuberculosis, tuberculosis-HIV coinfection, and those in tuberculosis retreatment. We used regression models using joinpoint regression for trend analysis, reporting the annual percentage change and the average annual percentage change.

**Results:**

For the three groups analyzed, we observed heterogeneity in the annual percentage change in the Brazilian FUs, with a predominance of significantly decreasing trends in the cure indicator in most FUs, especially at the end of the time series. When considering national indicators, an average annual percentage change of −0.97% (95% CI: −1.23 to −0.74) was identified for the cure of people with pulmonary tuberculosis, of −1.11% (95% CI: −1.42 to −0.85) for the cure of people with tuberculosis-HIV coinfection, and of −1.44% (95% CI: −1.62 to −1.31) for the cure of people in tuberculosis retreatment.

**Conclusions:**

The decreasing trends of cure indicators in Brazil are concerning and underscore a warning to public authorities, as it points to the possible occurrence of other treatment outcomes, such as treatment discontinuity and death. This finding contradicts current public health care policies and requires urgent strategies aiming to promote follow-up of patients during tuberculosis treatment in Brazil.

## INTRODUCTION

Despite its ancient origin and global efforts for control, tuberculosis remains an important public health problem.^1^ In 2022, the WHO estimated that 10.6 million individuals fell ill worldwide, and 1.3 million died from the disease.^1^ This scenario becomes even more complex and worrying when we consider that tuberculosis disproportionately affects underdeveloped and developing countries and the most socioeconomic vulnerable groups.^2^

Regarding this context, WHO launched the “End Tuberculosis Strategy”, an international proposal that aims to eliminate the global tuberculosis epidemic.^3^ This strategy is anchored in pillars of research and innovation, support systems, and integrated patient-centered care and prevention.^3^ The objectives are to reduce the incidence rate of tuberculosis by 90% and the number of deaths caused by the disease by 95% by 2035, according to data released in 2015.^3^

In relation to this issue, Sustainable Development Goals (SDGs) were also highlighted, which are directly related to combating tuberculosis as a public health problem. These include ending hunger and poverty, as well as reducing inequities, given that the disease has a strong social determination.^4^ In general, the SDGs presented by the United Nations aim to guarantee to the global population the end of persistent social inequalities, and environmental and climate protection.^4^

Brazil is one of the countries with the highest number of tuberculosis cases. As part of efforts to achieve global goals, the Ministry of Health launched the “National Plan to End Tuberculosis as a Public Health Problem”, which recognizes the WHO international pacts and the 2030 SDG agenda.^5^ However, recently, the COVID-19 pandemic caused negative effects on global health and reversed advances in tuberculosis control, including on a national level.^6^

Among the efforts towards the elimination of tuberculosis as a public health problem, the importance of actions aimed at linking individuals to health services for effective treatment follow-up stands out.^3^ This requires unique strategies focused on the specificities of individuals affected by the disease, with a focus on therapy adherence to increase cure rates, on reducing unfavorable outcomes (e.g., loss to follow-up and death), and controlling the transmission chain.^3^

However, a country with continental dimensions and regional inequalities, such as Brazil, implies the possibility of dissimilarity of indicators in geopolitical areas, which shape different actions and responses to tuberculosis.^5^ Therefore, with the aim of understanding the evolution of cure indicators in light of the history of actions already developed and identifying barriers towards achieving global goals, we analyzed the temporal trend of tuberculosis cure indicators in Brazil.

## METHODS

We conducted an ecological study that presented the Brazilian Federative Units (FUs) as the unit of analysis. We followed all the recommendations of the Reporting of Studies Conducted using Observational Routinely Collected Health Data (RECORD).^7^ Also, as this study used aggregated, anonymous, and publicly available data, it did not require an evaluation by a research ethics committee, in accordance with the Ordinance No. 674 of May 6, 2022, issued by the Brazilian National Health Council.

Brazil has over 200 million inhabitants and is stratified into 26 states and the *Distrito Federal*, organized into five regions: North, Northeast, South, Southeast, and Central-West. This organization ensures the adoption of different programmatic responses for the prevention and management of tuberculosis. The country has a gross domestic product per capita estimated at R$ 42,247.52, an unemployment rate of 7.7%, and an illiteracy rate among people over 15 years of age of 5.6%.^8^

We collected data from the Brazilian *Sistema de Informação de Agravos de Notificação* (SINAN, Notifiable Diseases Information System) via the *Departamento de Informática do Sistema Único de Saúde* (DATASUS, Information Technology Department of the Unified Health System), accessed in November of 2023. Regarding cases of tuberculosis, we should note that only people with a confirmed diagnosis are reported by health professionals at all levels of the health care network.^9^

For the study population, we included all tuberculosis cases reported between 2001 and 2022, considering that this period refers, respectively, to the initial availability of data on SINAN and the last year with qualified data until the date of access to DATASUS. Concerning the cure indicators studied, we considered the following definitions and formulas established in the recently released “Tuberculosis Indicators Booklet”, elaborated by the Brazilian Ministry of Health.^10^

- Percentage of cure among new cases of laboratory-confirmed pulmonary tuberculosis: the total number of laboratory-confirmed new cases of pulmonary tuberculosis that presented a status of closure as cure, in the year and locality, divided by the total number of new laboratory-confirmed cases of pulmonary tuberculosis in the year and locality, and then multiplied by 100;
- Percentage of cure among new cases of tuberculosis in people with HIV: the total number of new cases of tuberculosis among people with a positive test for HIV that presented a status of cure for tuberculosis, in the year and locality, divided by the total number of new tuberculosis cases among people with a positive test for HIV, in the year and locality, and then multiplied by 100;
- Percentage of cure of tuberculosis retreatment cases: the total number of tuberculosis retreatment cases cured, in the year and locality, divided by the total number of retreatment cases, in the year and locality, and then multiplied by 100.

We selected these indicators to explain tuberculosis cure over time in three different groups. Reported cases that had essential information (such as year of diagnosis, place of residence, and closure status) filled in as ignored or blank were not included, because these are data points do not allow a reliable analysis. We proceeded to data tabulation in an electronic spreadsheet (Microsoft Excel^®^), with indicators calculated according to the year of diagnosis and the FU of residence.^10^

Considering that a reduced number of events could generate important random variations in the proportion measurements, we decided to use the strategy of smoothing the historical series using the three-point moving average.^10^ Based on the initially calculated indicators, we averaged the values from the previous year, the current year, and the following year. This process resulted in series with 20 points (considered as the years after smoothing) for trend analysis.

We applied regression models via the Joinpoint Regression Program^®^, version 5.0 (National Cancer Institute, Bethesda, MD, USA), which aimed to visualize whether the insertion of several straight segments, verified by the identification of inflection points (joinpoints), is capable of better explaining the behavior of the set of data than are fewer segments or just one straight line.^11^ Based on the assumption defined in the literature,^11^ as 20 years were considered for the analysis, we postulated a maximum of three joinpoints per series.

The aforementioned indicators were defined as the dependent variable and, as the independent variable, the years of the historical series after smoothing (i.e., 2002-2021). To build the models, we applied the grid selection method, using the following parameters: natural logarithmic transformation of the dependent variable; adjustment of the models by the standard error of the percentages; and adjustment for first-order autocorrelation, verified by the “estimated from the data” function.^11^

The best model was the one with the lowest value in the weighted Bayesian criterion. We calculated the annual percentage change (APC), the average annual percentage change (AAPC), and corresponding 95% CIs using the empirical quantile method.^11^ The APC indicated the change in the segments, and the AAPC, which consists of the geometric mean of the APC, demonstrated the behavior across the entire series. The interpretation of the APC and AAPC was as follows:

- Increasing trend (↑): indicated by positive values of APC or AAPC with a 95% CI that did not encompass the null point (zero);
- Decreasing trend (↓): indicated by negative values of APC or AAPC with a 95% CI that did not encompass the null point (zero);
- Stationary trend (↔): characterized when the APC or AAPC, whether positive or negative, encompassed the null point (zero) within the 95% CI.

## RESULTS

Between 2001 and 2022, 994,081 new laboratory-confirmed cases of pulmonary tuberculosis were registered, of which 709,096 (71.33%) were declared cured. Regarding the trend of the cure indicator, we observed heterogeneity in the APC across FUs. We detected moments of increasing and decreasing trends throughout the analyzed period, but with a negative AAPC in most FUs (n = 24; 88.88%), except for Acre, Amapá, and Rio de Janeiro, as described in Table 1.

**Table 1:** Temporal trend in the cure percentage of new cases of laboratory-confirmed pulmonary tuberculosis in the Brazilian federative units between 2002 and 2021.

Federative Unit	Period	APC (95% CI)	Trend	AAPC (95% CI)	Trend
North region					
Rondônia	2002-2008	0.27 (−0.30;2.19)	↔	−1.55 (−1.90;−1.22)	↓
	2008-2012	−2.08 (−3.63;−0.89)	↓		
	2012-2019	0.79 (0.28;2.41)	↑		
	2019-2021	−13.22 (−16.57;−7.74)	↓		
Acre	2002-2007	3.03 (1.97;5.17)	↑	0.05 (−0.22;0.39)	↔
	2007-2019	0.29 (0.07;0.54)	↑		
	2019-2021	−8.39 (−10.67;−4.67)	↓		
Amazonas	2002-2004	−3.66 (−5.62;−0.86)	↓	−1.59 (−1.86;−1.33)	↓
	2004-2010	0.69 (0.16;2.16)	↑		
	2010-2019	−0.72 (−1.10;−0.39)	↓		
	2019-2021	−9.80 (−12.15;−6.12)	↓		
Roraima	2002-2013	−0.40 (−0.78;0.20)	↔	−1.68 (−2.08;−1.36)	↓
	2013-2016	−5.67 (−7.71;−2.82)	↓		
	2016-2019	3.84 (1.10;6.56)	↑		
	2019-2021	−10.25 (−14.53;−5.87)	↓		
Pará	2002-2013	0.17 (0.02;0.40)	↑	−1.23 (−1.46;−1.08)	↓
	2013-2016	−2.49 (−3.25;−1.36)	↓		
	2016-2019	2.67 (1.51;3.69)	↑		
	2019-2021	−12.05 (−14.33;−10.14)	↓		
Amapá	2002-2012	2.57 (1.15;7.83)	↑	0.38 (−0.17;1.14)	↔
	2012-2019	0.13 (−1.23;2.88)	↔		
	2019-2021	−9.07 (−14.57;−2.30)	↓		
Tocantins	2002-2007	2.16 (1.16;4.54)	↑	−1.06 (−1.52;−0.67)	↓
	2007-2019	−0.45 (−0.83;−0.10)	↓		
	2019-2021	−12.00 (−15.95;−6.79)	↓		
Northeast region					
Maranhão	2002-2007	1.12 (0.24;3.13)	↑	−0.85 (−1.16;−0.58)	↓
	2007-2016	−0.51 (−2.16;−0.04)	↓		
	2016-2019	1.08 (−0.25;2.27)	↔		
	2019-2021	−9.75 (−12.59;−5.82)	↓		
Piauí	2002-2004	−2.11 (−4.03;1.11)	↔	−1.58 (−1.91;−1.31)	↓
	2004-2007	3.09 (−0.70;4.19)	↔		
	2007-2018	−0.52 (−0.87;−0.16)	↓		
	2018-2021	−9.34 (−11.58;−6.50)	↓		
Ceará	2002-2006	4.04 (2.70;5.72)	↑	−1.52 (−1.95;−1.23)	↓
	2006-2019	−1.27 (−1.48;−1.07)	↓		
	2019-2021	−13.22 (−16.68;−8.29)	↓		
Rio Grande do Norte	2002-2021	−0.62 (−1.13;−0.11)	↓	−0.62 (−1.13;−0.11)	↓
Paraíba	2002-2021	−1.18 (−1.80;−0.65)	↓	−1.18 (−1.80;−0.65)	↓
Pernambuco	2002-2019	0.16 (−0.11;0.56)	↔	−0.99 (−1.52;−0.46)	↓
	2019-2021	−10.20 (−15.08;−3.45)	↓		
Alagoas	2002-2006	2.29 (0.81;5.31)	↑	−1.63 (−2.17;−1.11)	↓
	2006-2019	−1.64;(−2.03;−1.13)	↓		
	2019-2021	−9.02 (−13.54;−2.71)	↓		
Sergipe	2002-2012	−0.20 (−0.72;1.79)	↔	−1.26 (−1.81;−0.79)	↓
	2012-2016	−3.15 (−6.52;−1.23)	↓		
	2016-2019	4.59 (1.69;7.68)	↑		
	2019-2021	−10.76 (−16.33;−4.97)	↓		
Bahia	2002-2006	3.44 (2.47;5.05)	↑	−1.28 (−1.59;−0.99)	↓
	2006-2010	−1.40 (−2.45;−0.59)	↓		
	2010-2019	−0.29 (−0.57;0.78)	↔		
	2019-2021	−13.78 (−16.56;−8.72)	↓		
Southeast region					
Minas Gerais	2002-2008	1.41 (0.83;2.13)	↑	−1.36 (−1.64;−1.14)	↓
	2008-2016	−1.18 (−2.23;0.43)	↔		
	2016-2019	0.61 (−0.82;1.51)	↔		
	2019-2021	−12.53 (−15.11;−9.82)	↓		
Espírito Santo	2002-2006	1.34 (0.52;3.30)	↑	−2.75 (−3.19;−2.49)	↓
	2006-2016	−0.89 (−1.14;−0.53)	↓		
	2016-2019	−3.38 (−4.46;−1.45)	↓		
	2019-2021	−17.73 (−21.60;−13.06)	↓		
Rio de Janeiro	2002-2006	6.25 (4.37;9.84)	↑	0.32 (−0.07;0.70)	↔
	2006-2019	0.04 (−0.21;0.36)	↔		
	2019-2021	−8.98 (−12.04;−4.66)	↓		
São Paulo	2002-2012	0.55 (0.39;0.78)	↑	−0.95 (−1.12;−0.82)	↓
	2012-2019	−0.60 (−0.93;−0.33)	↓		
	2019-2021	−9.31 (−10.84;−7.93)	↓		
South region					
Paraná	2002-2004	−1.04 (−2.36;0.94)	↔	−1.84 (−2.16;−1.58)	↓
	2004-2013	1.00 (−1.49;2.08)	↔		
	2013-2019	−2.33 (−2.93;−1.59)	↓		
	2019-2021	−13.06 (−15.98;−8.88)	↓		
Santa Catarina	2002-2012	0.05 (−0.25;0.53)	↔	−1.55 (−1.91;−1.26)	↓
	2012-2019	−1.79 (−2.34;−1.06)	↓		
	2019-2021	−8.41 (−11.77;−4.23)	↓		
Rio Grande do Sul	2002-2006	1.00 (0.30;2.21)	↑	−1.75 (−1.99;−1.56)	↓
	2006-2011	−1.98 (−3.00;−1.42)	↓		
	2011-2019	−0.69 (−0.94;0.17)	↔		
	2019-2021	−10.44 (−12.68;−6.67)	↓		
Central-West region					
Mato Grosso do Sul	2002-2007	1.06 (0.44;1.90)	↑	−1.74 (−1.96;−1.57)	↓
	2007-2016	−1.93 (−2.38;−1.69)	↓		
	2016-2019	1.85 (0.51;2.70)	↑		
	2019-2021	−12.40 (−14.49;−10.47)	↓		
Mato Grosso	2002-2007	0.54 (−0.28;2.83)	↔	−1.60 (−1.99;−1.19)	↓
	2007-2010	−3.00 (−4.08;−1.35)	↓		
	2010-2019	−0.51 (−0.85;1.46)	↔		
	2019-2021	−9.33 (−13.24;−3.94)	↓		
Goiás	2002-2004	−2.96 (−5.97;1.30)	↔	−1.29 (−1.70;−0.90)	↓
	2004-2009	2.24 (−2.45;5.00)	↔		
	2009-2019	−1.01 (−1.46;0.42)	↔		
	2019-2021	−9.33 (−12.98;−4.80)	↓		
Distrito Federal	2002-2008	0.19 (−1.79;1.72)	↔	−4.28 (−4.98;−3.81)	↓
	2008-2015	−2.16 (−3.90;1.69)	↔		
	2015-2019	−8.82 (−10.13;−0.34)	↓		
	2019-2021	−14.82 (−21.01;−9.57)	↓		
Brazil	2002-2007	1.60 (0.94;2.78)	↑	−0.97 (−1.23;−0.74)	↓
	2007-2019	−0.47 (−0.67;−0.27)	↓		
	2019-2021	−9.88 (−12.14;−5.96)	↓		

APC: annual percentage change; AAPC: average annual percentage change; and (↑: increasing; ↔: stationary; and ↓: decreasing).

Concerning tuberculosis-HIV coinfection, we observed 143,361 cases during the study period, of which 69,794 (48.68%) were cured. It is noteworthy the divergence of APC across FUs, yet a declining trend at the conclusion of the series was observed for most states, except for Acre, Roraima, Tocantins, Rio Grande do Norte, and Mato Grosso. Acre, Amapá, and Rio de Janeiro exhibited an upward trajectory in AAPC, contrasting with the declining (n = 19; 70.37%) or stationary (n = 5; 18.51%) trends in the other FUs (Table 2).

**Table 2:** Temporal trend in the percentage of cure of new cases of tuberculosis with HIV coinfection in the Brazilian federative units between 2002 and 2021.

Federative Unit	Period	APC (95% CI)	Trend	AAPC (95% CI)	Trend
North region					
Rondônia	2002-2006	1.66 (−1.73;10.70)	↔	−1.25 (−1.98;−0.35)	↓
	2006-2010	−7.25 (−11.90;−3.22)	↓		
	2010-2019	2.23 (1.37;6.45)	↑		
	2019-2021	−9.59 (−16.02;−1.50)	↓		
Acre	2002-2006	19.24 (11.29;32.47)	↑	2.33 (1.42;3.90)	↑
	2006-2011	−8.89 (−15.53;−5.57)	↓		
	2011-2015	7.78 (3.36;14.51)	↑		
	2015-2021	−1.65 (−5.83;0.05)	↔		
Amazonas	2002-2005	−2.81 (−12.14;4.52)	↔	−0.84 (−1.48;0.04)	↔
	2005-2009	7.80 (−1.48;12.98)	↔		
	2009-2019	−1.27 (−1.72;−0.50)	↓		
	2019-2021	−11.63 (−15.29;−6.27)	↓		
Roraima	2002-2019	−1.14 (−7.57;24.79)	↔	−2.80 (−4.83;0.59)	↔
	2019-2021	−15.86 (−31.13;1.06)	↔		
Pará	2002-2004	−5.02 (−10.51;3.26)	↔	−1.56 (−2.11;−0.80)	↓
	2004-2010	4.16 (−2.40;9.04)	↔		
	2010-2019	−0.86 (−1.66;0.68)	↔		
	2019-2021	−16.57 (−20.28;−10.70)	↓		
Amapá	2002-2011	13.38 (12.20;16.46)	↑	2.48 (1.86;3.58)	↑
	2011-2014	−9.89 (−12.60;−4.49)	↓		
	2014-2018	3.69 (1.03;8.66)	↑		
	2018-2021	−15.27 (−21.40;−10.92)	↓		
Tocantins	2002-2004	25.95 (6.89;59.03)	↑	1.22 (0.98;3.65)	↔
	2004-2009	7.12 (−14.29;12.69)	↔		
	2009-2014	−11.00 (−17.66;6.91)	↔		
	2014-2021	0.10 (−13.58;9.51)	↔		
Northeast region					
Maranhão	2002-2019	0.22 (−1.23;7.07)	↔	−1.20 (−2.57;1.24)	↓
	2019-2021	−12.51 (−22.27;−0.08)	↓		
Piauí	2002-2012	0.77 (−4.73;8.74)	↔	−2.33 (−3.21;−1.13)	↓
	2012-2019	−2.89 (−5.27;4.50)	↔		
	2019-2021	−14.78 (−22.32;−3.93)	↓		
Ceará	2002-2004	15.10 (7.18;22.87)	↑	−1.69 (−2.21;−1.06)	↓
	2004-2007	−4.26 (−5.88;−1.24)	↓		
	2007-2018	−0.09 (−0.40;0.85)	↔		
	2018-2021	−14.34 (−16.43;−10.60)	↓		
Rio Grande do Norte	2002-2019	1.13 (0.38;30.49)	↑	−0.28 (−1.56;3.63)	↔
	2019-2021	−11.50 (−23.88;0.45)	↔		
Paraíba	2002-2011	−2.24 (−2.83;0.81)	↔	−3.03 (−3.74;−2.48)	↓
	2011-2014	−7.13 (−10.07;−3.72)	↓		
	2014-2017	6.21 (1.24;9.73)	↑		
	2017-2021	−8.11 (−14.54;−6.25)	↓		
Pernambuco	2002-2007	1.04 (0.03;2.92)	↑	−1.22 (−1.52;−0.94)	↓
	2007-2013	−2.47 (−4.21;−1.74)	↓		
	2013-2018	1.83 (0.78;3.81)	↑		
	2018-2021	−7.24 (−10.98;−4.84)	↓		
Alagoas	2002-2005	10.00 (2.19;23.84)	↑	−3.50 (−4.79;−2.05)	↓
	2005-2009	−13.32 (−18.85;−8.14)	↓		
	2009-2019	−0.73 (−1.91;7.18)	↔		
	2019-2021	−14.67 (−24.69;−3.83)	↓		
Sergipe	2002-2011	−0.35 (−1.59;2.84)	↔	−1.49 (−2.49;−0.54)	↓
	2011-2014	−12.69 (−17.62;−5.68)	↓		
	2014-2018	12.92 (8.44;23.54)	↑		
	2018-2021	−10.46 (−20.72;−4.81)	↓		
Bahia	2002-2007	10.00 (8.64;12.52)	↑	−0.11 (−0.54;0.36)	↔
	2007-2010	−5.81 (−7.03;−2.69)	↓		
	2010-2019	−0.59 (−0.99;0.50)	↔		
	2019-2021	−12.38 (−15.96;−7.29)	↓		
Southeast region					
Minas Gerais	2002-2011	2.17 (1.69;2.67)	↑	−0.52 (−0.91;−0.26)	↓
	2011-2016	−3.45 (−5.92;−2.45)	↓		
	2016-2019	2.88 (0.74;4.51)	↑		
	2019-2021	−9.61 (−13.40;−5.47)	↓		
Espírito Santo	2002-2010	1.65 (−0.75;3.29)	↔	−3.16 (−3.74;−2.72)	↓
	2010-2013	−3.65 (−5.32;3.43)	↔		
	2013-2018	0.22 (−1.61;3.28)	↔		
	2018-2021	−19.25 (−23.01;−14.80)	↓		
Rio de Janeiro	2002-2006	15.43 (13.72;17.78)	↑	2.25 (1.88;2.67)	↑
	2006-2010	−2.92 (−4.94;−1.32)	↓		
	2010-2019	1.41 (1.01;2.45)	↑		
	2019-2021	−7.61 (−10.77;−3.16)	↓		
São Paulo	2002-2008	0.23 (−2.21;1.24)	↔	−1.32 (−1.75;−1.02)	↓
	2008-2011	2.34 (−1.67;3.49)	↔		
	2011-2019	−0.48 (−1.35;1.06)	↔		
	2019-2021	−13.78 (−17.37;−8.57)	↓		
South region					
Paraná	2002-2010	2.40 (2.06;2.84)	↑	−1.49 (−1.73;−1.31)	↓
	2010-2016	−0.07 (−0.48;0.88)	↔		
	2016-2019	−2.34 (−3.20;−1.10)	↓		
	2019-2021	−18.07 (−20.41;−16.27)	↓		
Santa Catarina	2002-2012	1.70 (−1.25;5.62)	↔	−1.46 (−2.20;−0.82)	↓
	2012-2015	−5.11 (−7.29;4.15)	↔		
	2015-2019	−0.99 (−3.51;3.00)	↔		
	2019-2021	−11.74 (−18.84;−3.66)	↓		
Rio Grande do Sul	2002-2007	3.18 (2.42;3.82)	↑	−1.99 (−2.25;−1.79)	↓
	2007-2015	−2.11 (−2.83;−1.81)	↓		
	2015-2019	−0.79 (−1.65;0.33)	↔		
	2019-2021	−15.51 (−17.87;−13.30)	↓		
Central-West region					
Mato Grosso do Sul	2002-2008	−0.87 (−2.92;6.84)	↔	−3.93 (−5.31;−2.80)	↓
	2008-2011	−10.43 (−14.90;−4.37)	↓		
	2011-2019	1.22 (−0.41;11.14)	↔		
	2019-2021	−21.20 (−32.82;−9.74)	↓		
Mato Grosso	2002-2015	−3.73 (−7.45;4.39)	↔	−3.38 (−4.75;−1.63)	↓
	2015-2019	3.60 (−8.20;9.48)	↔		
	2019-2021	−13.96 (−26.26;0.21)	↔		
Goiás	2002-2011	−0.53 (−1.26;1.04)	↔	−1.92 (−2.60;−1.41)	↓
	2011-2014	−7.30 (−9.80;−3.50)	↓		
	2014-2018	4.25 (1.56;8.99)	↑		
	2018-2021	−8.30 (−17.10;−4.23)	↓		
Distrito Federal	2002-2015	−0.71 (−1.25;−0.08)	↓	−3.03 (−3.72;−2.62)	↓
	2015-2021	−7.88 (−11.15;−6.02)	↓		
Brazil	2002-2006	3.08 (1.88;5.24)	↑	−1.11 (−1.42;−0.85)	↓
	2006-2019	−0.54 (−0.74;−0.32)	↓		
	2019-2021	−12.37 (−14.84;−7.71)	↓		

APC: annual percentage change; AAPC: average annual percentage change; and (↑: increasing; ↔: stationary; and ↓: decreasing).

Regarding tuberculosis retreatment cases, we identified that, between 2001 and 2022, a total of 275,255 cases were registered, and 134,740 (48.95%) progressed to cure. We observed that, except for Rondônia, all FUs experienced a decreasing trend at the end of the historical series, particularly between 2019 and 2021. According to AAPC, we also detected a decreasing trend in the period for all FUs of analysis (n = 23; 85.18%), except for Acre, Amapá, Tocantins, and Rio de Janeiro (Table 3).

**Table 3:** Temporal trend in the cure percentage of tuberculosis retreatment cases in the Brazilian federative units between 2002 and 2021.

Federative Unit	Period	APC (95% CI)	Trend	AAPC (95% CI)	Trend
North region					
Rondônia	2002-2012	−1.42 (−10.20;12.92)	↔	−1.84 (−3.23;−0.38)	↓
	2012-2015	−7.85 (−15.35;9.57)	↔		
	2015-2019	8.34 (−6.85;20.06)	↔		
	2019-2021	−13.23 (−26.53;2.34)	↔		
Acre	2002-2019	1.67 (1.32;2.43)	↑	0.09 (−0.63;0.80)	↔
	2019-2021	−12.44 (−19.06;−5.52)	↓		
Amazonas	2002-2010	0.47 (−0.13;1.59)	↔	−2.18 (−2.46;−1.85)	↓
	2010-2013	−4.28 (−5.40;−2.21)	↓		
	2013-2019	−0.77 (−1.39;1.09)	↔		
	2019-2021	−13.06 (−15.78;−8.62)	↓		
Roraima	2002-2006	7.81 (4.27;11.51)	↑	−1.37 (−1.78;−0.83)	↓
	2006-2013	−10.37 (−12.74;−9.25)	↓		
	2013-2016	21.42 (14.66;25.66)	↑		
	2016-2021	−7.31 (−8.86;−5.91)	↓		
Pará	2002-2016	−0.51 (−2.32;−0.02)	↓	−1.33 (−1.92;−0.92)	↓
	2016-2019	4.70 (0.85;7.08)	↑		
	2019-2021	−14.81 (−20.04;−8.25)	↓		
Amapá	2002-2004	−12.65 (−25.59;6.93)	↔	0.05 (−0.83;1.68)	↔
	2004-2008	12.98 (−6.15;27.68)	↔		
	2008-2019	−0.15 (−1.32;6.35)	↔		
	2019-2021	−9.10 (−16.66;−1.83)	↓		
Tocantins	2002-2007	10.95 (2.49;26.19)	↑	−1.28 (−2.99;0.72)	↔
	2007-2019	−3.20 (−5.32;13.53)	↔		
	2019-2021	−17.01 (−29.86;−3.18)	↓		
Northeast region					
Maranhão	2002-2006	−0.38 (−1.39;1.96)	↔	−2.54 (−2.81;−2.29)	↓
	2006-2016	−2.10 (−3.72;−1.86)	↓		
	2016-2019	1.42 (−0.54;2.75)	↔		
	2019-2021	−14.07 (−16.63;−11.34)	↓		
Piauí	2002-2006	1.17 (−0.66;4.77)	↔	−1.67 (−2.09;−1.33)	↓
	2006-2011	−4.71 (−8.68;−3.18)	↓		
	2011-2014	5.36 (1.06;8.14)	↑		
	2014-2021	−3.94 (−5.90;−2.90)	↓		
Ceará	2002-2006	3.99 (2.73;6.21)	↑	−2.50 (−2.86;−2.20)	↓
	2006-2009	−4.35 (−5.35;−2.55)	↓		
	2009-2019	−2.19 (−2.49;−0.76)	↓		
	2019-2021	−13.17 (−16.42;−8.58)	↓		
Rio Grande do Norte	2002-2013	−4.45 (−4.73;−4.19)	↓	−1.74 (−1.99;−1.52)	↓
	2013-2019	6.55 (5.97;7.29)	↑		
	2019-2021	−10.16 (−12.56;−7.50)	↓		
Paraíba	2002-2006	−1.86 (−3.13;−0.01)	↓	−4.03 (−4.34;−3.81)	↓
	2006-2015	−6.78 (−7.32;−6.35)	↓		
	2015-2018	8.85 (6.93;10.39)	↑		
	2018-2021	−10.42 (−12.60;−8.64)	↓		
Pernambuco	2002-2007	0.24 (−1.55;5.54)	↔	−1.62 (−2.14;−1.09)	↓
	2007-2011	−2.85 (−5.67;2.28)	↔		
	2011-2019	1.74 (0.85;4.14)	↑		
	2019-2021	−15.89 (−20.55;−20.55)	↓		
Alagoas	2002-2007	−1.84 (−2.97;0.26)	↔	3.92 (−4.37;−3.56)	↓
	2007-2010	−10.49 (−12.60;−6.85)	↓		
	2010-2015	2.89 (0.95;8.06)	↑		
	2015-2021	−7.63 (−9.84;−5.98)	↓		
Sergipe	2002-2005	−8.93 (−12.78;−6.55)	↓	−2.98 (−3.41;−2.64)	↓
	2005-2011	−3.21 (−5.07;−1.27)	↓		
	2011-2019	0.28 (−0.26;3.25)	↔		
	2019-2021	−5.84 (−10.06;−1.49)	↓		
Bahia	2002-2019	−0.69 (−1.40;1.31)	↔	−2.53 (−4.15;−1.10)	↓
	2019-2021	−16.80 (−29.53;−2.00)	↓		
Southeast region					
Minas Gerais	2002-2007	0.78 (0.01;1.82)	↑	−2.07 (−2.40;−1.82)	↓
	2007-2015	−3.01 (−3.72;−2.59)	↓		
	2015-2019	2.94 (1.60;5.44)	↑		
	2019-2021	−14.26 (−17.39;−9.23)	↓		
Espírito Santo	2002-2017	0.19 (−0.87;0.64)	↔	−4.62 (−5.81;−3.95)	↓
	2017-2021	−19.57 (−25.35;−14.61)	↓		
Rio de Janeiro	2002-2005	4.03 (0.75;10.62)	↑	−0.41 (−1.03;0.19)	↔
	2005-2019	0.70 (−0.28;1.65)	↔		
	2019-2021	−13.71 (−18.56;−8.04)	↓		
São Paulo	2002-2005	3.31 (0.97;7.89)	↑	−0.86 (−1.22;−0.47)	↓
	2005-2019	0.18 (−0.24;0.44)	↔		
	2019-2021	−13.33 (−15.95;−8.17)	↓		
South region					
Paraná	2002-2004	−4.82 (−6.05;−2.45)	↓	−2.53 (−2.75;−2.33)	↓
	2004-2011	2.50 (2.09;3.14)	↑		
	2011-2019	−1.76 (−2.14;−1.42)	↓		
	2019-2021	−18.84 (−20.66;−17.17)	↓		
Santa Catarina	2002-2009	−1.12 (−4.90;0.30)	↔	−2.90 (−3.56;−2.43)	↓
	2009-2013	1.78 (−0.50;4.52)	↔		
	2013-2019	−3.72 (−5.27;−1.91)	↓		
	2019-2021	−14.97 (−20.30;−8.91)	↓		
Rio Grande do Sul	2002-2006	−0.34 (−1.56;2.03)	↔	−2.70 (−2.98;−2.42)	↓
	2006-2013	−3.55 (−5.08;−2.97)	↓		
	2013-2019	−0.08 (−0.74;1.33)	↔		
	2019-2021	−11.65 (−14.20;−7.47)	↓		
Central-West region					
Mato Grosso do Sul	2002-2011	0.89 (0.11;2.05)	↑	−2.54 (−3.01;−2.08)	↓
	2011-2014	−11.72 (−14.03;−6.97)	↓		
	2014-2019	4.88 (3.31;8.68)	↑		
	2019-2021	−19.49 (−23.65;−14.82)	↓		
Mato Grosso	2002-2008	−2.05 (−3.24;0.88)	↔	−2.87 (−3.37;−2.49)	↓
	2008-2011	−4.89 (−6.62;2.37)	↔		
	2011-2019	1.06 (0.03;2.91)	↑		
	2019-2021	−16.56 (−21.01;−10.41)	↓		
Goiás	2002-2007	1.89 (−3.25;10.55)	↔	−1.35 (−2.11;−0.48)	↓
	2007-2019	−1.31 (−3.62;5.44)	↔		
	2019-2021	−9.24 (−15.71;−1.45)	↓		
Distrito Federal	2002-2006	−6.00 (−10.84;−3.35)	↓	−5.40 (−6.32;−4.77)	↓
	2006-2010	7.93 (4.33;12.84)	↑		
	2010-2019	−7.20 (−8.03;−5.87)	↓		
	2019-2021	−19.75 (−26.84;−10.99)	↓		
Brazil	2002-2006	2.36 (1.78;3.41)	↑	−1.44 (−1.62;−1.31)	↓
	2006-2009	−2.77 (−3.32;−1.39)	↓		
	2009-2019	0.03 (−0.14;0.30)	↔		
	2019-2021	−13.99 (−14.92;−12.40)	↓		

APC: annual percentage change; AAPC: average annual percentage change; and (↑: increasing; ↔: stationary; and ↓: decreasing).

## DISCUSSION

Between 2002 and 2021, we identified a decreasing trend in cure indicators for new cases of pulmonary tuberculosis, cases of tuberculosis-HIV coinfection, and cases of retreatment in Brazil, with heterogeneity among the FUs. The reduction in the cure indicator is worrisome and issues a warning to public authorities, as it points to the possible occurrence of worse outcomes. Thus, these findings go against current public policies and underscore the need of immediate interventions in Brazil.

A time series analysis conducted in Brazilian capitals with data between 2001 and 2015 found significant heterogeneity in the temporal pattern of tuberculosis outcomes.^12^ Despite the disparity among locations, the authors showed an increasing trend of loss to follow-up during tuberculosis treatment in the country, while the cure rate remained stationary.^12^ This scenario already indicated difficulties related to improving adherence to treatment and cure rates in Brazil.

Similarly, a research conducted in the city of São Paulo between 2006 and 2017 pointed to an annual increase of 1.60% (95% CI, 0.02-3.48) in cases of treatment abandonment.^13^ Furthermore, a study in a state in the northeast of Brazil, analyzing over nine thousand tuberculosis cases, found a decreasing trend in the percentage of people cured, dropping from 81.78% in 2001 to 57.81% in 2016.^14^ This evidence corroborates and reinforces the findings obtained in this investigation.

Especially at the end of the time series, the effects of the COVID-19 pandemic can be attributed as one of the explanatory factors for the decreasing trends, which may also have influenced the general trend during the period. Researchers from several countries, such as Mozambique,^15^ Brazil,^16^ and Italy^17^ reported that the health situation that prevailed during the pandemic caused underdiagnosing and underreporting, an increase in treatment interruptions, and a reduction in the cure rates for tuberculosis.

In a scenario of national inequalities, the importance of targeting specific strategies to groups most socially vulnerable to the disease stands out. Considering the daily barriers that prevent adequate access to health services and the continuity of care,^18^ ^,^ ^19^ people deprived of liberty,^19^ people living with HIV, and people undergoing retreatment for the disease, for example,^20^ are more susceptible to the worst outcomes (e.g., loss to follow-up and death) of tuberculosis.

A meta-analysis investigating the results and predictors of tuberculosis treatment on the African continent observed that the treatment success rate varied between 53% (95% CI: 47-58%) in Nigeria and 92% (95% CI: 90-93%) in Ethiopia.^20^ The worst outcomes, such as death and loss of follow-up, were more associated with people living with HIV (relative risk [RR] = 1.53; 95% CI, 1.36-1.71) and people undergoing tuberculosis retreatment (RR = 1.48; 95% CI, 1.14-1.94).^20^

In the Brazilian context, a retrospective cohort study identified factors leading to loss of follow-up for tuberculosis treatment when compared with the cure outcome.^21^ Among the vulnerabilities, the following stood out: male gender (adjusted OR [aOR] = 1.35; 95% CI: 1.23-1.46); non-white ethnicity/race (aOR = 1.16; 95% CI: 1.07-1.26); drug use (aOR = 1.84; 95% CI: 1.66-2.04); and entry as recurrence (aOR = 1.33; 95% CI: 1.15-1.53) or re-entry after abandonment (aOR = 4.31; 95% CI: 3.90-4.77).^21^

To achieve the goals of reducing new cases and deaths from the disease by 2030, it is necessary and urgent to advance and overcome existing care gaps. To this end, we suggest expanding diagnostic testing, timely and adequate reporting of new cases, guaranteeing general access to health care for treatment follow-up, mitigation of disease risk factors (focusing on countries with the highest burden and among vulnerable populations), and expanding investments in research.^22^

The literature reported several useful strategies that focus on promoting care for people affected by tuberculosis that can be used to provide access to health services, adequate follow-up, and treatment. Directly observed treatment, for example, has been crucial to guarantee the bond between health professionals and affected people, allowing early identification of possible loss to follow-up and favoring the chances of curing the disease.^21^ ^,^ ^23^ ^,^ ^24^

Furthermore, to effectively combat tuberculosis, it is crucial to reinforce actions aimed at controlling and managing cases of latent *Mycobacterium tuberculosis* infection (LTBI), also known as tuberculosis infection.^25^ In these situations, people are not sick, but when exposed to risk factors, such as immunosuppression or malnutrition, they can develop an active condition, and, eventually, spread tuberculosis among contacts, maintaining the transmission chain.^26^

The Brazilian Ministry of Health has focused efforts on screening and treating cases of tuberculosis infection since 2018, when a surveillance protocol for latent *Mycobacterium tuberculosis* infection was published.^27^ A descriptive study that analyzed the indications for treatment of tuberculosis infection between January of 2018 and June of 2022 identified 85,822 cases of LTBI treatment in the country, especially among contacts of people with tuberculosis (57.2%) and people living with HIV (16.7%).^28^

In addition to the national plan that directs control strategies,^5^ the Brazilian Ministry of Health intensified the strengthening of actions that deal with social protection for people and families affected by tuberculosis, considering the close relationship between poverty and the disease.^29^ Intersectoral actions, focused mainly on education and social assistance, can be particularly important for ensuring comprehensive care and well-being for individuals and groups with tuberculosis.^20^

On this topic, in 2024, Decree No. 11,908 established the *Programa Brasil Saudável*, coordinated by the *Comitê Interministerial para a Eliminação da Tuberculose e de Outras Doenças Socialmente Determinadas*.^30^ The aim is to promote the integration of ministries, with the goal of quickly improving access to health care and mitigating existing inequalities in the country.^30^ One of the objectives is to eliminate tuberculosis as a public health problem by the end of this decade.^28^

However, given the worrying scenario of tuberculosis cure indicators in our study, we highlight the need to strengthen the health care network constantly, especially primary care. Primary health care has autonomy on the prevention, diagnosis, and treatment of tuberculosis.^31^ Also, the activities and coverage of primary health care teams are directly related to the detection of the disease in Brazil,^32^ playing a crucial role in monitoring cases and interrupting the transmission chain.

This study needs to be interpreted in light of some limitations: (i) the use of secondary data is subject to filling errors and underreporting, especially during the COVID-19 pandemic period; (ii) ignored/blank records about the outcome or location were excluded; (iii) there is the possibility of reviewing preliminary data between 2018 and 2022 by the Brazilian Ministry of Health; and (iv) the use of the percentage as a measure makes the dependent variable more susceptible to variations.

Therefore, it must be considered that the data employed in this study may not accurately reflect the actual scenario of the tuberculosis cure trend in the country. Undiagnosed, unreported, and/or inaccurately filled cases may overestimate or underestimate the calculated indicators. However, it is reiterated that smoothing strategies using a three-point moving average and the selection of complete cases were applied in an attempt to minimize these effects.

In short, this study highlighted the need for new efforts to mitigate the scenario of reduction in the cure rate among tuberculosis cases, especially in the most vulnerable populations. The coordination between health care services, social assistance, and other sectors of society is a key element in tackling this problem. Moreover, this is essential for achieving the goals agreed in the national plan for the elimination of tuberculosis by 2030, mainly in the post-pandemic period.

The need to evaluate local scenarios for monitoring tuberculosis indicators at a national level is highlighted, considering that there are particularities that lead to the adoption, or lack thereof, of different strategies for controlling the disease. In this sense, the results of our study are essential for managers, particularly those at a federal level, to understand the different epidemiological contexts and promote the redirecting of interventions to locations with the most alarming indicators.

## References

[1] World Health Organization (WHO) (c2023). Global tuberculosis report 2023.

[2] Maciel EL, Golub JE, Silva JRLE, Chaisson RE (2022). Tuberculosis a deadly and neglected disease in the COVID-19 era. J Bras Pneumol.

[3] World Health Organization (WHO) (c2014). The End TB Strategy.

[4] Nações Unidas Brasil Objetivos de desenvolvimento sustentável no Brasil.

[5] Brasil. Ministério da Saúde. Secretaria de Vigilância em Saúde. Departamento de Vigilância das Doenças Transmissíveis. Coordenação-Geral do Programa Nacional de Controle da Tuberculose (c2017). Brasil Livre da Tuberculose: Plano Nacional para a Eliminação da Tuberculose como Problema de Saúde Pública.

[6] Silva DR, Mello FCQ, Migliori GB (2022). Effects of COVID-19 on tuberculosis control past, present, and future. J Bras Pneumol.

[7] Benchimol EI, Smeeth L, Guttmann A, Harron K, Moher D, Petersen I (2015). The REporting of studies Conducted using Observational Routinely-collected health Data (RECORD) statement. PLoS Med.

[8] Brasil. Instituto Brasileiro de Geografia e Estatística (IBGE) (c2022). Cidades e Estados.

[9] Rocha MS, Bartholomay P, Cavalcante MV, Medeiros FC, Codenotti SB, Pelissari DM (2020). Notifiable Diseases Information System (SINAN) main features of tuberculosis notification and data analysis. Epidemiol Serv Saude.

[10] Brasil. Ministério da Saúde. REDE-TB (c2024). Caderno de indicadores da tuberculose: tuberculose sensível, tuberculose drogarresistente e tratamento preventivo.

[11] National Institutes of Health. National Cancer Institute (c2023). National Center Institute. Surveillance Research Program. Joinpoint Regression Program - version 5.0.2..

[12] Sousa GJB, Garces TS, Pereira MLD, Moreira TMM, Silveira GMD (2019). Temporal pattern of tuberculosis cure, mortality, and treatment abandonment in Brazilian capitals. Rev Lat Am Enfermagem.

[13] Berra TZ, Bruce ATI, Alves YM, Campoy LT, Arroyo LH, Crispim JA (2020). Related factors, time trend and spatial association of abandonment of treatment fortuberculosis in Ribeirão Preto-SP Rev. Eletr. Enferm.

[14] Lima SVMA, Dos Santos AD, Duque AM, de Oliveira Goes MA, da Silva Peixoto MV, da Conceição Araújo D (2019). Spatial and temporal analysis of tuberculosis in an area of social inequality in Northeast Brazil. BMC Public Health.

[15] Manhiça I, Augusto O, Sherr K, Cowan J, Cuco RM, Agostinho S (2022). COVID-19-related healthcare impacts an uncontrolled, segmented time-series analysis of tuberculosis diagnosis services in Mozambique, 2017-2020. BMJ Glob Health.

[16] Berra TZ, Ramos ACV, Alves YM, Tavares RBV, Tartaro AF, Nascimento MCD (2022). Impact of COVID-19 on Tuberculosis Indicators in Brazil A Time Series and Spatial Analysis Study. Trop Med Infect Dis.

[17] Di Gennaro F, Gualano G, Timelli L, Vittozzi P, Di Bari V, Libertone R (2021). Increase in Tuberculosis Diagnostic Delay during First Wave of the COVID-19 Pandemic Data from an Italian Infectious Disease Referral Hospital. Antibiotics (Basel).

[18] Pavinati G, Lima LV, Radovanovic CAT, Magnabosco GT (2023). Geoprogrammatic disparities in the performance of tuberculosis indicators in the homeless population in Brazil an ecological approach. Rev Bras Epidemiol.

[19] Macedo LR, Maciel ELN, Struchiner CJ (2021). Vulnerable populations and tuberculosis treatment outcomes in Brazil. Cien Saude Colet.

[20] Teferi MY, El-Khatib Z, Boltena MT, Andualem AT, Asamoah BO, Biru M (2021). uberculosis Treatment Outcome and Predictors in Africa: A Systematic Review and Meta-Analysis. Int J Environ Res Public Health.

[21] Lima LV, Pavinati G, Palmieri IGS, Vieira JP, Blasque JC, Higarashi IH (2023). Factors associated with loss to follow-up in tuberculosis treatment in Brazil a retrospective cohort study. Rev Gaucha Enferm.

[22] Floyd K, Glaziou P, Zumla A, Raviglione M (2018). The global tuberculosis epidemic and progress in care, prevention, and research an overview in year 3 of the End TB era. Lancet Respir Med.

[23] Zago PTN, Maffacciolli R, Riquinho DL, Kruse MHL, Rocha CMF (2022). Treatment adherence under the foucauldian perspective knowledge/powers in tuberculosis control manuals in Brazil. Rev Gaucha Enferm.

[24] Siqueira TC, Martellet MG, Tavernard GLN, Silva VM, Moura STS, Silva LAF (2020). Perception of nurses focus on the family and community orientation in tuberculosis actions. Cienc Cuid Saude.

[25] Brasil. Ministério da Saúde. Secretaria de Vigilância em Saúde (c2022). Protocolo de vigilância da infecção latente pelo Mycobacterium tuberculosis no Brasil.

[26] Chaw L, Chien LC, Wong J, Takahashi K, Koh D, Lin RT (2020). Global trends and gaps in research related to latent tuberculosis infection. BMC Public Health.

[27] Brasil. Ministério da Saúde (c2018). Protocolo de vigilância da infecção latente pelo Mycobacterium tuberculosis no Brasil.

[28] Pavinati G, Lima LV, Couto RM, Alves LC, Vega FLR, Silva DA (2023). Indicação do tratamento da tuberculose latente desafios identificados no sistema de notificação brasileiro, 2018-2022. Rev Saude Publica Parana.

[29] Brasil. Ministério da Saúde. Secretaria de Vigilância em Saúde (c2022). Guia Orientador: Promoção da Proteção Social para Pessoas Acometidas por Tuberculose.

[30] Brasil (2024). Presidência da República. Ministério da Casa Civil. Secretaria Geral para Assuntos Jurídicos.

[31] Rabelo JVC, Navarro PD, Carvalho WDS, Almeida IN, Oliveira CSF, Haddad JPA (2021). Performance assessment of primary healthcare services in tuberculosis control in a city in Southeast Brazil [Article in Portuguese] Cad Saude. Publica.

[32] Pelissari DM, Bartholomay P, Jacobs MG, Arakaki-Sanchez D, Anjos DSOD, Costa MLDS (2018). Offer of primary care services and detection of tuberculosis incidence in Brazil. Rev Saude Publica.

